# The development of a risk score for unplanned removal of peripherally inserted central catheter in newborns^1^

**DOI:** 10.1590/0104-1169.0491.2578

**Published:** 2015-07-03

**Authors:** Priscila Costa, Amélia Fumiko Kimura, Debra Huffman Brandon, Eny Dorea Paiva, Patricia Ponce de Camargo

**Affiliations:** 2 Doctoral student, Escola de Enfermagem, Universidade de São Paulo, São Paulo, SP, Brazil. RN, Escola de Enfermagem, Universidade de São Paulo, São Paulo, SP, Brazil; 3 PhD, Professor, Escola de Enfermagem, Universidade de São Paulo, São Paulo, SP, Brazil; 4 PhD, Associate Professor, School of Nursing, Duke University, Durham, NC, United States; 5 PhD, Professor, Escola de Enfermagem, Universidade Federal Fluminense, Rio de Janeiro, RJ, Brazil; 6 PhD, RN, Instituto da Criança, Hospital das Clínicas, Faculdade de Medicina, Universidade de São Paulo, São Paulo, SP, Brazil

**Keywords:** Catheterization, Central Venous, Risk Factors, Infant, Newborn, Neonatal Nursing

## Abstract

**OBJECTIVE:**

to develop a risk score for unplanned removal of peripherally inserted central catheter in newborns.

**METHOD:**

prospective cohort study conducted in a neonatal intensive care unit with newborn babies who underwent 524 catheter insertions. The clinical characteristics of the newborn, catheter insertion and intravenous therapy were tested as risk factors for the unplanned removal of catheters using bivariate analysis. The risk score was developed using logistic regression. Accuracy was internally validated based on the area under the Receiver Operating Characteristic curve.

**RESULTS:**

the risk score was made up of the following risk factors: transient metabolic disorders; previous insertion of catheter; use of a polyurethane double-lumen catheter; infusion of multiple intravenous solutions through a single-lumen catheter; and tip in a noncentral position. Newborns were classified into three categories of risk of unplanned removal: low (0 to 3 points), moderate (4 to 8 points), and high (≥ 9 points). Accuracy was 0.76.

**CONCLUSION:**

the adoption of evidence-based preventative strategies based on the classification and risk factors faced by the newborn is recommended to minimize the occurrence of unplanned removals.

## Introduction

Obtaining venous access in newborn babies admitted to neonatal intensive care units (NICUs) to infuse hyperosmolar, vesicant or irritating solutions is a challenge for nursing professionals. A peripherally inserted central catheter (PICC) is a central vascular access device placed at the bedside by a professionally qualified doctor or nurse, the tip of which is positioned close to the heart, preferably in the vena cava 1 ^-^ 2. Although the use of this device is increasingly frequent in NICUs, due to high insertion success rates and lower infection rates compared to surgically inserted central catheters^3^, studies carried out in Brazil show that rate of catheter-related complications range between 41 4 and 50.8%^5^, while international studies reveal lower rates, between 2.9^6^ and 31.7%^7^. Mechanical, thrombotic and infectious complications limit the effectiveness of PICCs and may lead to its removal earlier than planned. 

Studies with newborn babies which aimed to contribute towards preventing these complications and consequently reduce the occurrence of unscheduled PICC removal have identified a number of risk factors, including the insertion of the catheter through femoral veins^8^, spending more than sixty minutes on catheter insertion^9^, and non-central tip position^10^. However, the role of other potential risk factors among newborns, such as the clinical and anthropometric characteristics of the newborn, the type of catheter used and number of catheter lumens, the type of intravenous infusion in question, and previous PICC insertion history also merit investigation. 

Since the majority of catheter-related complications are preventable, the development of a risk score for unplanned PICC removal which considers the prognostic value of various risk factors is an innovative initiative for the advancement of nursing knowledge. Risk scores are potentially valuable tools for informing the decisions made by nurses, since they aid these professionals to estimate the likelihood of unplanned removal of bedside catheters prior to insertion, enabling case-by-case planning of care to attenuate risk. 

By developing a risk score for unplanned removal of peripherally inserted central catheters in newborns, this study therefore aims to contribute towards decreasing the prevalence of unplanned catheter removal and the suffering of newborns and their family caused by PICC-related complications, and to reducing hospital costs resulting from repeated catheter insertions and prolongation of the hospitalization of newborns. In addition, it seeks to generate information to guide evidence-based nursing interventions and consequently improve the quality of nursing care in NICUs.

## Method

This investigation comprises a prospective cohort study involving the collection of observational data from the medical records of newborn babies that underwent intravenous therapy through a PICC during the period 31 August 2010 to 30 August 2012 in the NICU of a private hospital in the municipality of São Paulo, Brazil. The project was approved by the hospital's ethics committee (Nº 238/2010).

Based on a previous study^11^ conducted in the same NICU which observed that the prevalence of unscheduled PICC removal was 37.7%, the minimum odds ratios which could be detected for a binary stratification variable with the sample size used in this study (524 PICC insertions), at the 5% level of significance, with 80% power, was 1.45.

The sample included infants born in the hospital's maternity ward who undergone the insertion of a PICC without the use of any other type of central venous access. The following exclusion criteria were used: absence of information in the medical record regarding the cause of the removal of the PICC; and the occurrence of death or transfer of the newborn while the PICC remained in situ.

The management of PICCs in this institution follows the guidelines set out in an institutional protocol designed by nurses from the venous catheters study group, based on the literature^12^^-^ 13 and recommendations given by institutions accredited to provide capacity building on the insertion, maintenance and removal of PICCs by the Regional^2^ or Federal Council of Nursing 14. PICC insertion must be recommended by a doctor after an assessment of the newborn's clinical condition and venous network and is an aseptic procedure which is conducted at the bedside by a qualified nurse. The medical and nursing teams use radiograph of the posterior and anterior aspects of the chest to determine the positioning of the tip of the device. The catheter is handled using sterile gloves and 70% alcohol swabs to disinfect the connections of the closed system. The PICC is permeabilised with saline solution before and after the infusion of intravenous medication. The dressing is changed using a standardised aseptic technique as and when necessary, when the transparent film loses its adhesion or when there is excessive bleeding in the insertion site. 

Data was registered using a pre-prepared form containing relevant study variables: clinical diagnosis, sex, postnatal age, gestational age and weight on the data of the procedure, type of catheter used (1.9 French silicone catheter, or 2.0 polyurethane double-lumen catheter), insertion site, position of the tip of the catheter (central or noncentral), number of intravenous solutions indicated for catheter insertion, number of previous PICC insertions, the length of time the catheter remained in situ, and date and motive for removal. Scheduled PICC removal was defined as that occurring at the end of infusion therapy or due to the prescription of solutions which are compatible with peripheral administration. The removal of a catheter was defined as unplanned when it was due to complications such as obstruction, rupture, tip migration, phlebitis, thrombosis, catheter-related bloodstream infection, swelling, infiltration, leakage, and accidental catheter removal. 

Data was stored in an excel spreadsheet using double entry and analysed in the R 3.01 environment. After applying the eligibility criteria, the data from 80% of the cases of PICC insertion was used to develop the risk score, while the data from the remaining 20% of cases was used for the internal validation of the risk score. First, the quantitative variables were analysed using averages and standard deviation. Qualitative variables were also analysed to ascertain the absolute and relative frequency distribution. Bivariate analysis was conducted to ascertain whether there was an association between variables and the outcome (unscheduled removal of PICC) using the Student t-test for continuous variables, the Chi-squared test or Fisher's exact test for categorical variables, and the estimation of relevant risk and 95% confidence interval. The significance level was set at 5%. The risk score was developed by conducting stepwise logistic regression using forward selection with the variables which were shown to have a significant association under the bivariate analysis. Only statistically significant and noncollinear variables were retained. The risk score was constructed based on the magnitude of correlation of the coefficients of each variable in the logistic equation. The predictive capacity of the score was evaluated based upon the area below the Receiver Operating Characteristic curve (ROC curve). The points of the ROC curve were used to construct three risk categories for unscheduled PICC removal: low, medium and high risk. For internal validation of the tool, the risk score was applied to the data reserved for validation to evaluate its predictive capacity in relation to the outcome based on the absolute and relative frequency distribution of the three risk categories.

## Results

A total of 17,341 infants were born during the study period, of which 1,482 were admitted to the NICU. Of this total, 460 underwent intravenous therapy, resulting in a total of 563 PICC insertions. After exclusion based on study eligibility criteria, the sample was reduced to 436 newborns who underwent a total 524 PICCs which was divided into two data sets: data used to develop the risk score (80% of the PICCs = 419); and data used for the initial validation of the risk score (20% the PICCs =105). 

The majority of the newborns were male (55.2%). Corrected average gestational and postnatal age were 33.7 weeks and 9.4 days, respectively, while average weight was 1,833.6 grams. The main clinical problems experienced by the newborns were premature birth (82.6%) and respiratory distress (68.3%). The majority of newborns (80%) had not undergone PICC insertion before. The most common type of device used was the polyurethane double-lumen catheter, which was used in 53.8% of the cases, and the most commonly used insertion site was the upper limb (72.6%). The majority of catheters (85.5%) were centrally placed in anatomical positions such as the superior vena cava and cavoatrial junction. The catheters were recommended for an average of 3.18 intravenous solutions, the majority of which were antibacterial (76.3%), followed by parenteral nutrition (66,8%).

The majority of PICCs (62.8%) were removed as planned at the end of intravenous therapy. However, 195 (37.2%) catheters were removed because of complications, of which the most common was catheter-related bloodstream infections (13.5%), followed by obstruction (5.9%), accidental removal (5.1%), external rupture (4.8%), leakage (2.1%), swelling of the limb (1.9%), phlebitis (1.7%), spontaneous migration of the catheter (1.3%), infiltration (0.4%), cardiac tamponade (0.2%), and thrombosis (0.2%). The catheter remained in situ for an average of 11.8 days (range of one to 70 days). 

The results of the analysis of the association between the outcome in question, unscheduled PICC removal, and the variables related to the clinical and anthropometric characteristics of the newborns, PICC insertion procedure, and the recommended intravenous therapy that led to the use of a catheter are shown in Table 1.

**Table 1.** Distribution of risk factors for unplanned removal of peripherally inserted central catheters in newborn babies. São Paulo, Brazil 2010 to 2012

Risk factors		Unplanned removal		Relative risk (95% confidence interval)	p value
		Yes (N=156)	No (N=263)		
Weight	≤1500 g	80(51.9%)	102(39.2%)	1.35[1.07-1.76]	0.01
	>1500g	74(48.1%)	158(60.8%)		
Gestational age	≤32 weeks	71(45.5%)	82(31.2%)	1.78[0.66-0.93]	0.004
	>32 weeks	85(54.4%)	181(68.8%)		
Postnatal age	≤7 days	93(59.6%)	224(85.2%)	1.84[1.41-2.38]	<0.001
	>7 days	63(40.4%)	39(14.8%)		
Clinical diagnosis					
Sepsis	Yes	38(24.4%)	40(15.4%)	1.26[1.0-1.59]	0.03
	No	118(75.6%)	220(84.4%)		
Heart disease	Yes	35(22.4%)	30(11.5%)	1.41[1.08-1.86]	0.005
	No	121(76.6%)	230(88.5%)		
Transient metabolic disorder	Yes	22(14.1%)	9(3.5%)	2.24[1.28-3.91]	<0.001
	No	134(85.9%)	251(96.4%)		
Previous insertion of PICC*	Yes	52(33.3%)	29(11%)	1.93[1.43-2.61]	<0.001
	No	104(66.7%)	234(89%)		
Insertion site	Upper limb	109(71.7%)	190(73.6%)	Reference	0.65
	Lower limb	22(14.5%)	41(15.9%)	0.95[0.66-1.38]	
	cervical region	16(10.5%)	18(7%)	1.29[0.87-1.90]	
	Cephalic region	5(3.3%)	9(3.5%)	0.97[0.47-2]	
Polyurethane double-lumen catheter	Yes	96(61.5%)	128(49.2%)	1.20[1.03-1.39]	0.001
	No	60(38.5%)	132(50.8%)		
Catheter tip in noncentral position	Yes	32(21.2%)	28(11%)	1.40[1.06-1.86]	0.008
	No	119(78.8%)	227(89%)		
Number of intravenous solutions –	Average (standard deviation)	3,47(1.73)	2.99(1.5)		0.003

*PICC = peripherally inserted central catheter

Table 1 shows that there was an association between unplanned removal of catheters and the following variables: weight ≤ 1500g, corrected gestational age ≤ 32 weeks, postnatal age > 7 days, early or late clinical diagnosis of sepsis, heart disease (persistent arterial duct, pervious foramen ovale, ventricular septal and atrial septal defects), transient metabolic disorders (hypoglycaemia, hyperglycaemia, calcium, magnesium, sodium and potassium imbalances), previous insertion of PICC, use of a polyurethane double-lumen catheter, tip in a noncentral position, and PICC recommended for an average of three intravenous solutions. A multivariate analysis was performed of the statistically significant and noncollinear variables in order to estimate the probability of unscheduled removal due to the risk factors identified by the bivariate analysis. Table 2 shows the variables which remained associated after logistic regression. 

**Table 2.** Risk factors for unscheduled removal of peripherally inserted central catheter in newborn babies identified by logistic regression. São Paulo, Brazil 2010 to 2012

Risk factor	β Coefficient	Standard error	Z value	P value	Odds ratio [95%Confidence interval]
Intercept	-2.08	0.39	-5.31	<0.001	
One or more previous insertions	1.36	0.28	4.93	<0.001	3.89 [2.28 – 6.74]
Transient metabolic disorders	1.51	0.43	3.51	<0.001	4.52 [2.00- 10.99]
Number of solutions (continuous)	0.27	0.12	2.21	0.03	1.30 [1.03 – 1.67]
PICC*Polyurethane double-lumen catheter	1.39	0.53	2.63	0.01	4.02 [1.44 – 11.54]
Noncentral position of tip	0.74	0.30	2.46	0.01	2.10 [1.16 – 3.82]

*PICC= peripherally inserted central catheter

**Figure 1.**
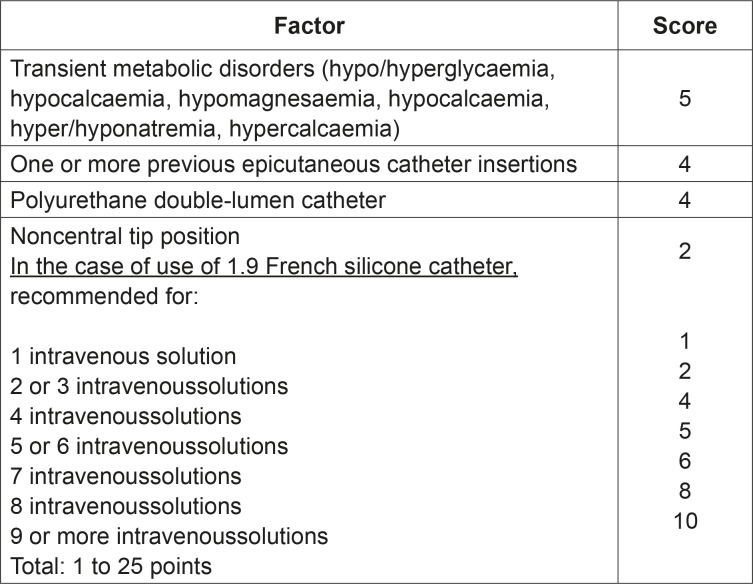
Risk score for unplanned removal of peripherally inserted central catheter in newborns . São Paulo, Brazil 2010 to 2012

The risk score was constructed according to the odds ratio of each explicative variable (Figure 1). Values were rounded to the nearest whole number to make up a simplified risk score which is usable for nurses in their everyday practice. An association was observed between number of intravenous solutions and type of PICC. The likelihood of unplanned removal of silicone catheters increased substantially when PICCs were recommended for five or more intravenous solutions, while the likelihood of unplanned removal of polyurethane double-lumen catheters remained practically constant regardless of the number of intravenous solutions.

The accuracy of the risk score, i.e., its predictive capacity for unscheduled PICC removal was evaluated using the area under the ROC curve (Area: 0.76 [CI 95%: 0.73-0.78). The initial validation of the simplified risk score was performed using the validation database (N = 105 PICCs) considering three risk cut-off points for unscheduled removal (low, moderate and high risk). The results showed that 26.1% of the catheters classified as low risk, 36% of those classified as moderate risk, and 64% of those classified as high risk were removed before planned.

## Discussion

The use of new technology such as PICCs for intravenous therapy may contribute to an increase in the survival rate of premature and severely ill babies admitted to NICUs. The identification of new risk factors and the development of a risk score for unplanned removal of PICCs among this group has an important role to play in planning nursing care directed at preventing common complications which lead to the unplanned removal of catheters, and also serves to help nurses detect the initial signs of these complications. 

The area under the ROC curve verified that the risk score developed by this study has good predictive capacity for unplanned PICC removal. These results are similar to the findings of a study which identified the risk factors and develop a predictive score for invasive candidiasis among infants (area under the ROC curve = 0.764 [CI 95%: 0.719-0.809)^15^. 

The study observed that the likelihood of complications that lead to the unplanned removal of catheters was almost five-times greater among newborns with transient metabolic disorders than in infants who did not have this disorder. Other studies show that the prevalence of transient metabolic disorders such as hyperglycaemia, hypercalcaemia, and hypoglycaemia in low birth weight babies was 57%^16^, 26.7%^17^and 8.7%^18^, respectively. Newborns with some form of transient metabolic disorder may therefore be more susceptible to PICC-associated bloodstream infections, since they are likely to need more frequent changes of intravenous solutions infused through PICCs due to their unstable condition. Therefore, strategies to prevent the unplanned removal of PICC newborns with this type of disorder should focus on catheter handling techniques.

Studies show that the use of checklists of evidence-based practices, standard care procedures, bundles, and a team dedicated to PICC care were associated with a reduction in complications, particularly catheter-related bloodstream infections^19^^-^ 20. The following procedures help to prevent this type of complication: continuing education for health professionals that handle and manage catheters on a daily basis; the use of aseptic techniques and maximal sterile barrier precautions, such as sterile gloves, gowns, head covers, and surgical mask covering the nose and mouth, and surgical field covering the newborn's body when inserting the PICC; ultrasound-guided venipuncture to reduce the number of puncture attempts and mechanical complications associated with insertion; the use of transparent bio-occlusive dressings to protect the insertion site; daily assessment of the need of the catheter; and catheter connection antisepsis before every use^21^^-^ 22.

Other mechanical complications such as obstruction, rupture and accidental removal are also preventable. A systematic review concluded that the use of heparin in PICCs in doses of 0.5 IU/kg/hr to prevent complications reduces the occurrence of obstruction, thus allowing a higher number of newborn babies to complete intravenous therapy^23^. However, another study which evaluated 188 PICCs inserted in newborn babies revealed that the complication rate was higher in infants who received continuous infusion of heparin than in those who did not (23.7/1,000 catheter days versus 17.2/1,000 catheter days)^24^. Given the fact that complications in newborn babies such as haemorrhaging, thrombocytopenia, and bleeding disorders may be related to continuous heparin infusion ^23^, conclusive evidence to the contrary is required to support this practice. 

PICC dressing-related procedures are an important element in the prevention of accidental removal, rupture of the external portion of the catheter and infection, and should follow certain principles such as avoiding excessive handling of the catheter and changing the dressing only when it is soiled or when it is loose and the insertion site is exposed^21^. 

Another risk factor was having experienced previous PICC insertions. Similar results were found by a study which analysed 1,524 PICC insertions in children, showing that the prevalence of catheter-related complications was greater (P <0,0001) in successive insertions^25^. The prevention of this risk factor includes avoiding unnecessary PICC removal to guarantee the functioning of the catheter until it is no longer necessary.

This study showed that the risk of unplanned PICC removal was twice as great when the tip was in a noncentral position. A retrospective cohort study carried out in a NICU in Canada which included 319 newborn babies observed similar results, showing that the risk of complications was 3.8 times greater when the PICC was inserted in the midclavicular region and 1.47 times greater when inserted in the brachiocephalic vein in comparison to PICCs inserted in the superior vena cava^10^. A preventative strategy for this risk factor is the accurate measurement of the length of the catheter, i.e., the distance between the puncture site to the vena cava along the vein^1^, together with close monitoring of the newborn for initial signs of complications such as infiltration^10^.

Another risk factor was the type of catheter used: findings suggest that risk of unscheduled removal was four times greater with polyurethane double-lumen catheters than with single-lumen silicone catheters. However evidence showing which material is best is not conclusive. A study compared the silicone catheter with an anti-reflux valve and the polyurethane PICC without a valve in 26 adults and concluded that the prevalence of complications between the two groups was similar^26^. However, the occurrence of complications is influenced not only by the material, but also the number of lumens. A study which analysed 4,000 PICC placements in adults using 4 Fr single-lumen and 5 Fr double-lumen catheters in a Canadian hospital concluded that the catheter replacement rate, costs and PICC-related bloodstream infection rate fell after the implementation of a policy to stimulate the use of single-lumen silicone PICCs in outpatients and inpatients where vascular access was required to infuse antibiotics, or where there were only few options for venous access 27.

Apart from the tip of the PICC, it is necessary to consider the number of solutions for which the catheter is required. Medication interaction in the catheter lumen, particularly in single-lumen catheters used for the infusion of multiple intravenous solutions, and also in double-lumen catheters with a single end hole for two routes, may lead to an increase in the occurrence of complications such as obstruction and rupture. Similar results were found by a study which analysed the frequency and type of complication in 610 PICCs used to administer antibiotics in children. The complication rate of epicutaneous catheters used to administer up to four daily doses of antibiotics was 16.2/1,000 catheter days, and 23.6/1000 catheter days for those used to administer over four doses. The relative risk of complications was 1.45 times greater for catheters used to administer over four daily doses 28. The prevention of complications in newborn babies receiving multiple endovenous solutions through PICCs includes careful maintenance of the catheter to prevent infection, obstruction and rupture.

The risk score developed by this study helps to provide accurate information so that health professionals are better able to identify the individual risk for each newborn and define the necessary strategies to prevent complications during catheter insertion and during the period in which the catheter remains in situ, and also comprises a useful tool to promote systematic clinical reasoning in nurses. It comprises an innovative method to estimate the risk of this undesired outcome which is applicable at the bedside. However, it is important to highlight that nursing interventions should be evidence-based and it is crucial to act on the risk factors identified for each newborn baby in order to reduce the occurrence of common complications which lead to unscheduled PICC removal, such as catheter-related bloodstream infections, obstruction and accidental removal.

Although this study involved a cohort of 524 epicutaneous catheter insertions, it has certain limitations. The study was restricted to only one private hospital in the city of São Paulo and therefore the risk factors and risk score portray the practices of the health professionals of this institution and the characteristics of the babies born in the maternity ward during the period in question, which may compromise the generalisation of data to other populations. However, the results observed by this study were congruent with the findings of other studies.

## Conclusion

The risk score for unplanned PICC removal developed by this study is a potentially useful tool for the identification of risk factors and for the classification of the individual risk of unplanned catheter removal among newborns. In this sense, these findings seek to guide the adoption of evidence-based preventative strategies in order to minimize the occurrence of unplanned removals of catheters associated with common complications such as catheter-related bloodstream infection, obstruction, and accidental removal.
